# Acceptability and feasibility of HIV self-testing integration into publicly-funded HIV prevention services: Perspectives from HIV testing agency staff that provide HIV testing services to sexual and gender minority youth in Philadelphia County

**DOI:** 10.1371/journal.pone.0320290

**Published:** 2025-03-25

**Authors:** Dovie L. Watson, Stephen Bonett, Steven Meanley, Sarah M. Wood, Kathleen A. Brady, José A. Bauermeister

**Affiliations:** 1 Department of Medicine, Division of Infectious Diseases, University of Pennsylvania Perelman School of Medicine, Philadelphia, Pennsylvania, United States of America; 2 Department of Family and Community Health, University of Pennsylvania School of Nursing, Philadelphia, Pennsylvania, United States of America; 3 Craig Dalsimer Division of Adolescent Medicine, Children’s Hospital of Philadelphia, Philadelphia, Pennsylvania, United States of America; 4 Department of Pediatrics, University of Pennsylvania Perelman School of Medicine, Philadelphia, Pennsylvania, United States of America; 5 Philadelphia Department of Public Health, Philadelphia, Pennsylvania, United States of America; University of Zimbabwe Faculty of Medicine: University of Zimbabwe College of Health Sciences, ZIMBABWE

## Abstract

**Background:**

Increasing HIV testing among priority populations is a primary strategy of the Ending the HIV Epidemic initiative. In October 2019, the Philadelphia Department of Public Health (PDPH) established a program to distribute publicly-funded HIV self-testing (HIVST) kits to Philadelphia County residents aged 16 years and older.

**Methods:**

Through a community-academic partnership, we used a cross-sectional sequential transformative mixed-methods design to examine perceived organizational factors, opportunities, and challenges to HIVST integration among agency staff at PDPH-funded agencies early in the COVID-19 pandemic due to decreased access to traditional in-person HIV testing services with a focus on agencies whose client populations included sexual and gender minority clients assigned male sex birth aged 13 to 24 years (not the sole population served at each agency). We integrated data from online surveys conducted with HIV testers (test counselors and testing leads), agency leaders (agency leads and directors), and care navigators (n =  42), and semi-structured interviews with HIV testers and agency leaders (n =  11) employed at PDPH-funded agencies.

**Results:**

Many staff were familiar with HIVST (79%), and approximately two-thirds (64%) were likely to encourage HIVST to clients. In interviews, perceived benefits of HIVST integration were increased access to HIV testing, accommodation for client privacy, decreased risk of stigmatizing encounters, and testing program adaptability. Perceived challenges were loss of connection with clients, suboptimal linkage to HIV treatment and prevention services after self-testing, concerns regarding clients’ correct use or interpretation of test results, and client preference.

**Conclusions:**

Agency staff described HIVST as a useful tool for expanding low-barrier HIV testing services; however, staff foresaw potential implementation challenges. To optimize HIVST as a long-term strategy, resources are needed to increase familiarity and comfort with HIVST and enhance staff’s capacity to establish meaningful client connections and link clients to post-test HIV treatment and pre-exposure prophylaxis services.

## Introduction

The United States Centers of Disease Control and Prevention (CDC) estimated that 13% of the nearly 2 million people living with HIV in 2019 had not yet received a formal HIV diagnosis, with undiagnosed individuals living with HIV accounting for 38% of new HIV transmissions [1,2]. Similar suboptimal rates of HIV status awareness were shown in Philadelphia County where the Philadelphia Department of Public Health (PDPH) estimated 11.2% of people living with HIV in Philadelphia were unaware of their status, and those unaware of their HIV status accounted for 39% of new HIV transmissions [3]. As one of the Ending the HIV Epidemic (EHE) priority jurisdictions, which together account for more than 50% of HIV transmissions in the United States, Philadelphia County was allocated additional federal funding and resources to expand the local strategies as outlined by the Philadelphia Department of Public Health EHE Community Plan to diagnose all persons living with HIV as early as possible [3–6]. As the entry point for status-neutral care (i.e., HIV treatment or pre-exposure prophylaxis (PrEP) services), increasing HIV status awareness will be critical to achieve population-level reductions in new HIV infections [7–11]. However, there are enduring inequities in HIV testing due to numerous barriers including poverty, limited access to health care, intersectional stigma, and capital disinvestment in racially segregated neighborhoods [12–14].

In addition, there was a pronounced decline in the number of HIV screening tests performed nationally early in the COVID-19 pandemic due to shortages in HIV testing materials and substantial disruption in HIV testing and service infrastructure [15]. In Philadelphia specifically, there was a decline in the number of HIV screening tests performed using PDPH-funded community and mobile-based testing, clinical testing, and prison-based testing—from an average volume of 7,000 screening tests per month (January 2019 to January 2020) to less than 5,000 screening tests per month (March 2020 to December 2020) [6]. Innovative strategies like HIV self-testing (HIVST) seek to minimize these barriers by expanding HIV testing beyond traditional health care settings [10,16].

Frontline staff and leadership at community-based HIV testing agencies are critical informants to understanding the challenges and opportunities for implementing strategies for HIV testing, counseling, and linkage to care. Research conducted prior to the COVID-19 pandemic found HIVST was highly acceptable, increased testing frequency, and expanded testing to priority populations that were sub-optimally engaged (e.g., cisgender sexual minority men, transgender people, and sex workers) [17–25]. However, the acceptability of HIVST with accompanying telehealth services among staff at community-based HIV testing organizations in the United States since the onset of the COVID-19 pandemic has not been well characterized.

In October 2019, the Division of HIV Health at the Philadelphia Department of Public Health launched the HIV home testing program through the “*Philly Keep on Loving*” website followed by partnerships with community-based PDPH-funded HIV testing and prevention service agencies to distribute publicly-funded HIVST kits to Philadelphia County residents [8]. Through the HIV home testing program, publicly-funded HIVST kits were made available to any Philadelphia resident aged 16 years and older—regardless of the individual’s self-reported HIV risk behavior, race, ethnicity, gender, or sexual orientation. Many agencies tailored their services to at least one specific priority population in Philadelphia, such as sexual and gender minority populations, Black or Latine communities, or people who inject drugs [26]. Philadelphia residents may request a publicly-funded HIVST kit through the HIV home testing program by one of the following mechanisms: (1) the resident may submit an online request to the Philadelphia Department of Public Health directly via the “*Philly Keep on Loving*” website and receive the kit to the mailing address provided; (2) the resident may request and pick up a kit directly from a community-based partner; or (3) the resident may request a kit from the community-based partner, who will submit an online request on the resident’s behalf, and the resident will receive the kit to the mailing address provided [3,8]. If kit recipients inform the community-based partner about their HIVST results, staff utilize their agency-specific procedures to link the individual to post-test HIV prevention or treatment services. Among agencies that provide HIV treatment and/or prevention services within their organizations, staff may link clients to internal confirmatory HIV testing and services. Among agencies without these internal services, the Philadelphia Department of Public Health provides technical assistance for frontline staff to coordinate confirmatory testing and linkage to external services in collaboration with other state and local governmental agencies. The health department also maintains and monitors Participating Provider Agreements with community-based partners to ensure systematic linkage to those services [26]. Early in the COVID-19 pandemic, the number of publicly-funded HIVST kits distributed to community-based partner agencies through the HIV home testing program increased each year, with 715 kits distributed in 2020, 1388 kits distributed in 2021, and 1762 kits distributed in 2022 [3]. Of note, minimal data collection regarding HIVST kit recipients was required by the Philadelphia Department of Public Health to promote low-barrier access to HIV testing services.

In October 2019, the Division of HIV Health also launched a community-academic implementation trial with the University of Pennsylvania Center for AIDS Research (Penn CFAR) [27]. The primary aim of the parent mixed-method implementation trial was to collect and analyze data using the original Consolidated Framework for Implementation Research (CFIR) [28] to systematically identify system-level determinants of HIV prevention service delivery in Philadelphia (i.e., the CFIR Outer Setting domain) and develop an intervention to optimize HIV prevention services at HIV testing agencies (i.e., the CFIR Inner Setting domain) whose client populations include young sexual and gender minority (SGM) individuals assigned male sex at birth aged 13 to 24 years (not the sole population served at each agency). The parent implementation trial included survey and interview questions about the “HIV Home Testing Program.” The objective of this secondary analysis was to examine perceived opportunities and challenges related to HIVST integration among frontline staff and leadership at PDPH-funded HIV testing agencies in Philadelphia County.

## Methods

### Study design and setting

**Fig 1:**
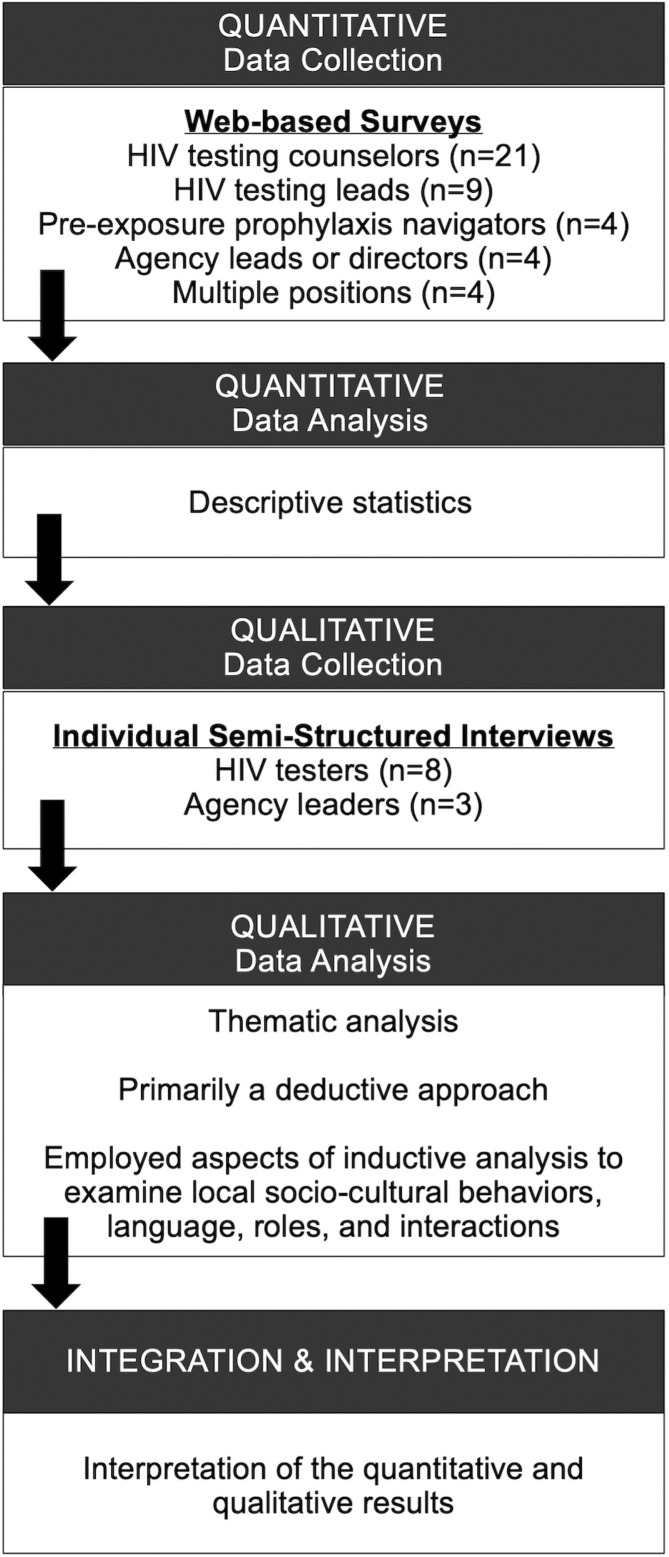
Analytic procedure for the sequential transformative mixed-methods evaluation.

In the planning phase of the parent implementation trial, we utilized the original Consolidated Framework for Implementation Research [28] and a sequential transformative mixed-methods design (QUANT◊QUAL), which involved two distinct data collection phases with their results integrated in the final data interpretation (see **Fig 1**) [29–31]. The cross-sectional data for this secondary analysis come from the planning phase of the ongoing implementation partnership between the Philadelphia Department of Public Health and the Penn CFAR, and procedures have been previously detailed [27]. Briefly, community-based service organizations were eligible to participate in the implementation trial if the organization (1) provided HIV testing, sexually transmitted infection (STI) testing, and/or PrEP services in Philadelphia; (2) self-identified as serving youth aged 13 to 24 years (not required to be the sole or the predominant client population served by the agency); and (3) received funding from the Philadelphia Department of Public Health to increase HIV testing and PrEP service delivery. Agency staff from enrollment organizations were recruited to participate in the implementation trial between 4 October 2020 and 2 November 2020 and were eligible to participate if they met the following criteria: (1) worked at a PDPH-funded HIV testing agency located in Philadelphia; (2) aged 18 years or older; (3) agreed to consent to study activities; and (4) planned to stay employed at their agency over the next 12 months. All study protocols and procedures involving human subjects were approved by the Institutional Review Boards of the Philadelphia Department of Public Health and University of Pennsylvania and performed in accordance with the ethical standards as laid down in the 1964 Declaration of Helsinki and its later amendments. Written informed consent was obtained from all participants prior to initiating study activities.

### Quantitative data collection

The study began with a 15-minute, confidential, voluntary web-based survey conducted with frontline agency staff and agency leaders between 5 October 2020 and 3 November 2020 via Qualtrics (Qualtrics, Provo, UT). The Philadelphia Department of Public Health provided agency rosters with staff titles and duties, and the Penn CFAR research team emailed invitations to complete the survey to agency staff. The authors based the survey questions on the domains included in local and National HIV Behavioral Surveillance guidelines for HIV counseling and testing certification and were written for a seventh-grade literacy level (see S1 File). The survey included items related to respondents’ sociodemographic characteristics; work experiences; sexual health knowledge (e.g., HIV/STI testing, PrEP, post-exposure prophylaxis, and HIV/STI treatment); knowledge, attitudes, and perceptions related to HIVST and telehealth services; and self-efficacy to perform typical duties related to HIV/PrEP and comprehensive sexual health service delivery (e.g., HIV test counseling, PrEP counseling, linkage to HIV medical care, and PrEP navigation) [27].

Survey respondents were asked if their agencies offered HIVST kits to individuals who accessed services provided by the organization (i.e., clients; yes or no). Questions regarding (1) familiarity with HIVST; (2) HIVST relevance to their agency’s outreach activities; (3) HIVST usefulness as a complement to the agency’s HIV testing capacity; (4) perceived ability to provide telehealth HIVST services; (5) perceived ability to complete post-test counseling during telehealth sessions; and (6) perceived ability to link clients to post-test HIV prevention and treatment services during telehealth sessions were presented on a 4-point Likert scale. Questions regarding the degree to which respondents (1) would encourage HIVST; (2) felt comfortable communicating with clients about HIVST; and (3) felt HIVST would be useful to agency clients, be simple to integrate, and support more frequent HIV testing among their clients were presented on a 5-point Likert scale. Questions regarding the degree to which respondents felt authentic/genuine and confident conducting telehealth sessions were presented on a 3-point Likert scale. The question related to the respondents’ perception of their clients’ experience during telehealth sessions was presented on a 5-point Likert scale. Respondents were asked to select their most common challenges related to telehealth HIV test counseling with an option to provide free-text responses. Survey respondents received a $20 electronic gift card as compensation for completing the survey.

### Qualitative data collection

Interviews were conducted between 18 November 2020 and 18 December 2020 with HIV testers and agency leaders at PDPH-funded HIV testing agencies enrolled in the implementation trial. We used the original Consolidated Framework for Implementation Research [28] and a qualitative descriptive research design to inform qualitative data collection. Qualitative description is a widely cited approach in nursing and healthcare research that offers broad insight into a particular health care-related phenomenon (e.g., HIV self-testing) and participants’ experiences and perceptions to develop interventions within a specific healthcare setting (e.g., publicly-funded HIV testing agencies) [32–36]. We recruited agency leaders and HIV testers to participate in interviews using purposive sampling to ensure inclusion of agency staff who did not complete the survey. In the parent implementation trial, we conducted individual interviews with both HIV testers and agency leaders to ensure variability in perspectives and enable researchers to obtain a comprehensive understanding of agency staff’s experiences related to HIV testing and prevention service provision. Semi-structured interview guides (see S2 File) included open-ended questions to examine perceived barriers and facilitators to PrEP implementation, HIVST integration, and lessons learned for future scalability. Topics included job duties, experiences, and perceived contextual factors related to HIV testing and prevention service provision. Interviews were conducted through teleconference, audio-recorded, and lasted 45-60 minutes. Participants received a $20 electronic gift card as compensation for completing the interview. There were a limited number of staff working in specific roles within the participating agencies; therefore, we did not collect sociodemographic information from interview participants to minimize the risk of potential identification and confidentiality breach.

### Data analysis

Given the exploratory nature of this study, we computed descriptive statistics using frequencies with percentages for categorical data and medians with interquartile ranges for continuous measures. Quantitative data were analyzed using Stata version 18 (StataCorp LLC, College Station, TX, 2023). Our qualitative analysis relied primarily on a deductive approach informed by the original Consolidated Framework for Implementation Research [37]. We also employed aspects of inductive analysis to examine local socio-cultural behaviors, language, roles, and interactions that might impact future planning and implementation of the intervention. Interview audio files were professionally transcribed verbatim. Authors DW and SB developed an initial codebook addressing multiple implementation determinants based on the original Consolidated Framework for Implementation Research [28] and implementation strategies for improving HIV prevention services. The coding team met to iteratively revise the codebook and discussed coding discrepancies to reach consensus. Authors SB and SM further examined data related to HIVST and developed an additional codebook specific to the research questions around HIVST, which was used to apply additional codes to excerpts of interest. Authors DW and SB conducted thematic analysis to generate and refine themes that might provide more detailed information about interviewees’ beliefs and perspectives on HIVST integration at their agencies. Qualitative data were analyzed using Dedoose (Version 9.0.86, Los Angeles, CA).

### Integration of findings

Key findings from our analysis of quantitative survey data from frontline agency staff and agency leaders and qualitative individual interviews with HIV testers and agency leaders were organized into a matrix [38–40]. We focused on integration and synthesis of key results from both methods to identify factors within the Intervention Characteristics, Outer Setting, and Inner Setting domains of the original Consolidated Framework for Implementation Research [37] that may be targeted in a systems-level intervention to improve publicly funded HIVST services from the standpoint of the health department. Equal weight was given to qualitative and quantitative data sources [38].

## Results

### Quantitative survey

Among the 80 eligible agency staff across 18 PDPH-funded HIV testing agencies who were invited to participate in the survey, 42 staff across 13 agencies completed the survey (56.2% response rate). Among these 13 agencies, 11 were community-based HIV service organizations, and two were academically affiliated medical practices that serve people living with HIV and those seeking HIV prevention services. Sociodemographic characteristics for the 42 survey respondents are provided in **Table 1**. Respondents’ median age was 36 years [interquartile range 30.5 – 51 years]. Most respondents self-identified as cisgender (98%), 45% as LGBTQ + , 55% as non-Hispanic/non-Latine Black, 21% as Hispanic/Latine, and 19% as a non-Hispanic/non-Latine person of another race. More than 70% reported they were full-time salaried employees (76%), primarily held an HIV test counselor role or HIV testing lead role (50% and 21%, respectively), and had at least 5 years of experience working in HIV services (81%). Approximately 60% reported at least 5 years of experience working as an HIV tester, specifically.

**TABLE 1:** Sociodemographic characteristics of HIV testing agency staff who completed online survey, Philadelphia County, 2020 (N =  42).

Characteristic	Median [Interquartile range]	n	(%)
Age, range [21 to 70 years]	36 (30.5-51)		
Race and ethnicity			
Non-Hispanic/Non-Latine Black or African American		23	54.8
Non-Hispanic/Non-Latine Another race		8	19.0
Hispanic or Latine, Any race		9	21.4
No response		2	4.8
Gender identity			
Cisgender man		19	45.2
Cisgender woman		22	52.4
Transgender person		1	2.4
Sexual orientation			
Heterosexual		23	54.8
Lesbian, gay, bisexual, queer, questioning, and sexual identities not listed		19	45.2
Position type at agency			
Full-time salaried		32	76.2
Part-time or hourly		10	23.8
Current position at agency			
HIV testing counselor		21	50.0
HIV testing lead		9	21.4
Pre-exposure prophylaxis navigator		4	9.5
Agency lead or director (Agency leaders)		4	9.5
Multiple positions		4	9.5
Length of time working as HIV tester			
Less than 5 years		16	38.0
5-10 years		14	33.3
More than 10 years		11	26.2
Length of time working in HIV services			
Less than 5 years		8	19.0
5-10 years		19	45.2
More than 10 years		15	35.7

Percentages may not total 100 due to rounding. Race and ethnicity were self-reported via confidential survey and categorized by investigators based on the US Office of Management and Budget’s Revisions to the Standards for the Classification of Federal Data on Race and Ethnicity. Respondents who self-reported as neither Black nor Hispanic were combined into the “Non-Hispanic, Another race” category to preserve anonymity due to small sample sizes.

More than half of the survey respondents reported that their agency offered to mail HIVST kits to their clients’ homes (25; 60%). As shown in **Table 2**, more than three-quarters of agency staff were somewhat or very familiar with HIVST (33; 79%); more than two-thirds reported that HIVST was very or extremely relevant to the services conducted at their agencies (30; 71%); and two-thirds reported HIVST was a very or extremely useful complement to the HIV testing services provided by their agencies (28; 67%).

**Table 2:** HIV self-testing service delivery familiarity, relevance, and usefulness among HIV testing agency staff, Philadelphia County, 2020 (*N* =  42).

	Not at all n (%)	Vaguely n (%)	Somewhat n (%)	Very n (%)	Median [IQR]
Familiarity with HIV self-testing kits ^a^	4 (9.5)	5 (11.9)	16 (38.1)	17 (40.5)	3 (3-4)
	**Not at all** **n (%)**	**Somewhat** **n (%)**	**Very** **n (%)**	**Extremely** **n (%)**	**Median** **[IQR]**
Relevance of HIV self-testing as related to HIV prevention outreach activities conducted at the agency ^b^	4 (9.5)	8 (19.0)	21 (50.0)	9 (21.4)	3 (2-3)
Usefulness of HIV self-testing implementation as a complement to the agency’s HIV testing capacity ^c^	4 (9.5)	10 (23.8)	16 (38.1)	12 (28.6)	3 (2-4)

**Abbreviations:** IQR =  interquartile range.

Percentages may not total 100 due to rounding or participant’s lack of response.

^a^Likert score: 1 =  Not at all familiar; 2 =  vaguely familiar; 3 =  somewhat familiar; 4 very familiar.

^b^Likert score: 1 =  Not at all relevant; 2 =  somewhat relevant; 3 =  very relevant; 4 =  extremely relevant.

^c^Likert score: 1 =  Not at all useful; 2 =  somewhat useful; 3 =  very useful; 4 =  extremely useful.

Almost two-thirds of agency staff (27; 64%) agreed or strongly agreed that they would encourage the use of HIVST, and HIVST would improve access to HIV prevention services among the client population served by their agencies (see **Table 3**). More than three-quarters of agency staff agreed or strongly agreed that they would feel comfortable communicating with clients about using an HIVST kit (33; 79%). Two-thirds of agency staff (28; 67%) agreed or strongly agreed that HIVST would save clients’ time traveling to HIV prevention services, would be simple to integrate into the agency’s testing capacity, and would be simple to integrate into test counselors’ duties at their agencies. However, less than half of agency staff agreed or strongly agreed that it would be simple for the clients served by their agency to integrate HIVST as a testing option (19; 45%), and more than one-fifth of agency staff disagreed or strongly disagreed that providing kits would ensure clients regularly completed HIV testing (9; 22%).

**Table 3:** HIV self-testing service provision willingness and perceived usefulness, ease, and acceptability of HIV self-testing among HIV testing agency staff, Philadelphia County, 2020 (*N* =  42).

	Strongly Disagree n (%)	Disagree n (%)	Neutral n (%)	Agree n (%)	Strongly Agree n (%)	Median [IQR]
I would encourage the use of an HIVST kit to test my clients. ^a^	3 (7.1)	3 (7.1)	9 (21.4)	16 (38.1)	11 (26.2)	4 (3-4.75)
An HIVST kit will improve clients’ access to HIV prevention services. ^a^	1 (2.4)	4 (9.5)	10 (23.8)	14 (33.3)	13 (31.0)	4 (3-5)
I would feel comfortable communicating with a client about using an HIVST kit. ^a^	2 (4.8)	1 (2.4)	6 (14.3)	18 (42.9)	15 (35.7)	4 (4-5)
An HIVST kit will save clients’ time traveling to HIV prevention services. ^a^	1 (2.4)	4 (9.5)	9 (21.4)	13 (31.0)	15 (35.7)	4 (3-5)
An HIVST kit will be simple to integrate into my agency’s testing capacity. ^a^	3 (7.1)	3 (7.1)	8 (19.0)	14 (33.3)	14 (33.3)	4 (3-5)
An HIVST kit will be simple to integrate into my responsibilities as a test counselor. ^a^	3 (7.1)	3 (7.1)	8 (19.0)	14 (33.3)	14 (33.3)	4 (3-5)
An HIVST kit will be simple for clients to integrate as an HIV testing option. ^a^	2 (4.8)	4 (9.5)	17 (40.5)	8 (19.0)	11 (26.2)	3 (3-4.75)
An HIVST kit will ensure that clients get tested regularly. ^a^	2 (4.8)	7 (16.7)	10 (23.8)	14 (33.3)	9 (21.4)	4 (3-4)

**Abbreviations:** HIVST = HIV self-testing; IQR = interquartile range

The term “clients” refers to Philadelphia residents who accessed services at the publicly-funded HIV testing agencies enrolled in the parent implementation trial. Percentages may not total 100 due to rounding or participant’s lack of response.

^a^Likert score: 1 =  Strongly disagree; 2 =  disagree; 3 =  neutral; 4 =  agree; 5 =  strongly agree; all items were reverse coded from original scale.

More than 80% of agency staff reported that it would be fairly easy or very easy to show their clients how to use an HIVST kit during a telehealth session (see **Table 4**). More than 70% reported that it would be fairly easy or very easy to complete post-test counseling (29; 77%), link an agency client to HIV care (28; 72%), and link an agency client to PrEP care (30; 77%) during a telehealth session.

**Table 4:** Perceived difficulty in HIV self-testing service provision and telehealth HIV services among HIV testing agency staff, Philadelphia County, 2020 (*N* =  39).

	Very hard n (%)	Fairly hard n (%)	Fairly easy n (%)	Very easy n (%)	Median [IQR]
Show a client how to use an HIVST kit during a telehealth session a	1 (2.6)	6 (15.4)	16 (41.0)	16 (41.0)	3 (3-4)
Complete post-test HIV prevention counseling during a session visit a	3 (7.7)	6 (15.4)	14 (35.9)	16 (41.0)	3 (3-4)
Link a client to HIV care during a telehealth session a	4 (10.3)	7 (17.9)	15 (38.5)	13 (33.3)	3 (2-4)
Link a client to PrEP care during a telehealth session a	3 (7.7)	6 (15.4)	14 (35.9)	16 (41.0)	3 (3-4)

**Abbreviations:** HIVST = HIV self-testing; IQR = interquartile range

The term “client” refers to a Philadelphia resident who accessed services at the publicly-funded HIV testing agencies enrolled in the parent implementation trial. Percentages may not total 100 due to rounding or participant’s lack of response.

^a^Likert score: 1 =  Very hard; 2 =  fairly hard; 3 =  fairly easy; 4 =  very easy; all items were reverse coded from original scale.

**Fig 2:**
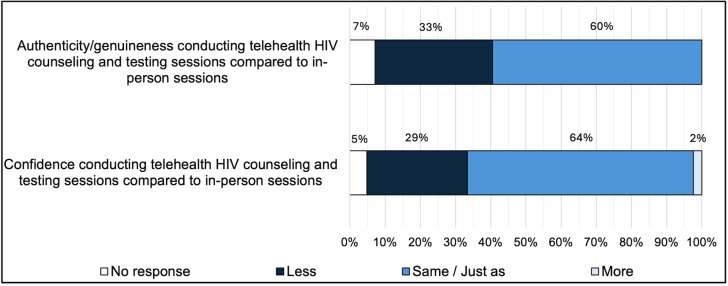
Perceived confidence and authenticity/genuineness conducting telehealth sessions among staff at publicly funded HIV testing agencies, Philadelphia County, 2020 (N=  42).

**Fig 3:**
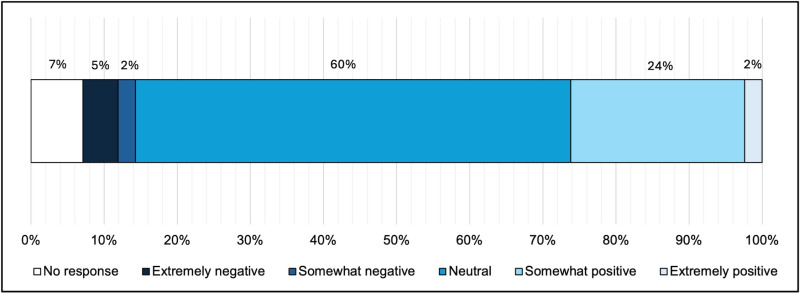
Perceived clients’ experience with HIV counseling and testing during telehealth sessions among staff at publicly funded HIV testing agencies, Philadelphia County, 2020 (N=  42).

Compared to in-person sessions, more than half of agency staff (25; 60%) reported feeling the same degree of authenticity/genuineness when interacting with clients during telehealth sessions, and two-thirds of agency staff (28; 66%) reported feeling the same degree of confidence or more confident conducting HIV counseling and testing during telehealth sessions (see **Fig 2**). Less than 10% of agency staff perceived that clients’ telehealth experiences were somewhat or extremely negative (see **Fig 3**).

**Fig 4:**
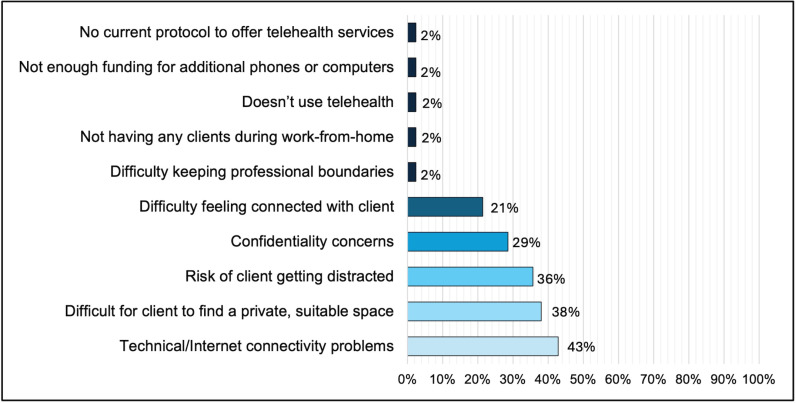
Main challenges conducting telehealth HIV test counseling visits among HIV testing agency staff. Listed items were not mutually exclusive, thus percentages total more than 100 percent.

As shown in **Fig 4**, the most commonly reported challenges to conducting HIV test counseling during telehealth sessions were technical/internet connectivity problems (43%), clients’ difficulty finding a private space to conduct the session (38%), risk of client distraction (36%), confidentiality concerns (29%), and difficulty feeling connected with clients (21%). Percentage totals more than 100 percent because responses were not mutually exclusive.

### Qualitative interviews

Among the 37 HIV testers and agency leaders invited to participate in an interview, 8 out of 27 HIV testers and 3 out of 10 agency leaders from seven PDPH-funded HIV testing agencies agreed to participate and discussed their experiences with HIVST during interviews (response rate 30% for each group). Six of the agencies were community-based HIV service organizations, and one was an academically affiliated medical practice that served people with HIV and those seeking HIV prevention services. **Table 5** summarizes the main themes that HIV testers and agency leaders discussed during individual interviews regarding key perceived advantages and disadvantages to HIVST integration.

**Table 5:** Summary of the main themes from individual interviews with HIV testers and agency leaders regarding HIV self-test (HIVST) integration into existing HIV testing programs.

Perceived benefits and advantages	Perceived challenges and disadvantages
HIVST integration supports sustainable access to low-barrier HIV testing services.	Standard operating procedures should be consistently implemented to track HIVST kit recipients and link them to local post-test HIV treatment and prevention services.
HIVST mitigates clients’ concerns about discretion and lack of trust in traditional in-person HIV testing services.	Clients may have challenges correctly using HIVST kits or interpreting self-test results at home.
HIVST decreases the risk of potential stigmatizing experiences related to HIV testing.	Some clients may strongly prefer traditional in-person HIV testing venues rather than self-test at home.

The main themes, main findings, and illustrative quotes of the interviewees’ views regarding HIVST integration into existing HIV testing programs are presented in **Table 6** and the six main themes are individually summarized below.

**Table 6:** Main themes from individual interviews about HIV self-testing (HIVST) integration with HIV testers and agency leaders (n = 11).

Main Themes	Main Findings	Illustrative Quotes
HIVST integration supports sustainable low-barrier HIV testing services.	HIVST may increase access to testing due to convenience and the option to mail publicly funded kits directly to clients’ homes.	• “I think it’s the convenience of having that [HIVST kit]. They don’t have to wait for an appointment. They just get it, and they do it by themselves at home.” (Participant 5, HIV tester) • “I think for some populations that wouldn’t get tested any other way, I think that is an effective strategy […] Those kits are expensive […] (Participant 2, Agency leader)
	HIVST may extend local testing service delivery due to secondary distribution of kits.	• “People been using the HIV test kits; however, not necessarily the person that picked it up or the person that the tests are being mailed to that use the test. I see a lot of people that are requesting tests, but they give to their partner, their mother, their father, the next-door neighbor…‘I’m just calling to follow up on the home test kit.’ ‘Oh, I gave the test to my neighbor.’” (Participant 7, HIV tester)
	Agencies were able to adapt testing programs early in the COVID-19 pandemic.	• “We’ve been [self-testing] for a couple months now, and it’s very promising because a lot of people are scared to go to doctors right now because of COVID-19. And we know this is a dangerous time for people when they want to interact, especially in the young [men who have sex with men] community.“(Participant 9, HIV tester) • “And with COVID-19 still going on we were still able to do a [branded outreach program] [...] it brought the clientele we were looking for. And because you can’t do [on-site] HIV tests, we were able to get the city to authorize the [HIVST] test. And that approach was the easiest way to reach the city’s numbers. We will literally have 30, 40, 50 people at the [branded outreach program] and they will take HIV test kits home.” (Participant 4, Agency leader)
	HIVST integration helped agencies meet testing metrics set by external funders.	• “People came in to be tested and the reality was we were not meeting our numbers. And the reality is not a lot of the organizations were reaching their numbers. But the home tests, I have trepidations with the home test, but the home test allowed us to have a connection.” (Participant 4, Agency leader)
HIVST mitigates concerns about discretion and lack of trust in traditional HIV testing services.	HIVST may increase testing among clients who do not trust the health care system.	• “Like, this [HIVST] is big…in underserved communities where they feel like they’ve very uncomfortable, and they don’t trust the health care system. And it’s been very promising for my organization because not only have people been taking these tests, but they’ve been reaching back out to us and telling us their results.” (Participant 9, HIV tester)
	HIVST may make it easier to accommodate clients’ need for discretion and privacy.	• “People became more interested in [HIVST], and they actually are a hit for us now…A lot of people have been interested in them because they can just [test] in the safety of their home.” (Participant 1, HIV tester)
HIVST decreases potential stigmatizing experiences related to HIV testing.	HIVST may decrease the risk of stigmatizing health care encounters with agency staff.	• “These home tests where people can go in the privacy of their own home, and they don’t have to worry about that stigma of going into the doctor’s office and have to explain to them that you know, ‘This is the reason why I want the test.’” (Participant 9, HIV tester)
	HIVST may decrease the risk of experiencing community-level HIV stigma.	• “They don’t have to worry about walking in [to the agency] and possibly being seen by somebody they may know.” (Participant 1, HIV tester)
Standard operating procedures (SOPs) should be more consistently implemented to track HIVST kit recipients and monitor HIVST results.	Staff at agencies that implemented SOPs to monitor clients expressed greater comfort and enthusiasm with HIVST integration.	• “Now, when somebody is interested in a [HIVST] kit we can mail it to them, or they can come pick it up in your office. And I do a follow-up after like two weeks after we mail it or two weeks after they pick up to see the test results. If it’s negative, [I] give information regarding HIV, HIV prevention, PrEP, and if it’s positive, connect to our medical care. If the person is negative and they’re interested in PrEP, I also connect to medical care.” (Participant 7, HIV tester)
	Staff were concerned about potential loss of connection with kit recipients due to minimal data collection.	• “We lose that connection once [clients] take the test home. You aren’t actually required to write anything down, but my staff and I are asking them, let’s find a way to keep connected with that individual, so we’re processing a way to keep connection with [recipients].” (Participant 4, Agency leader)
Clients may have challenges correctly completing HIVST at home.	Clients may experience challenges correctly using the test.	• “I’m like, ‘Wait. What is the follow up?’ […] We have to able to explain exactly what they need to do, how they need to do it, you know, in order to be safe and to make sure they’re doing it the correct way. […] Cause some people don’t even feel comfortable doing that way [self-testing] or they can’t understand the directions.” (Participant 6, HIV tester)
	Clients may experience challenges correctly interpreting results.	• “I got a client that it was educated, has an insurance, and he got the reactive result and didn’t believe it that it was reactive. And he came to get tested for the second time […] And still, when I give the results of a reactive, the client didn’t believe it. Then I realized after asking questions and things, I realized that the client didn’t really know that it was reactive.” (Participant 5, HIV tester)
Some individuals may strongly prefer traditional on-site HIV testing over HIVST.	Universal integration among publicly-funded HIV testing agencies may be impeded due to concerns about suboptimal linkage to post-test services.	• “I have mixed feelings on [HIVST]. I mean we choose not to do that as an organization. We just feel like too many people get lost. And they might take the test, and if they’re positive, what if they don’t go for care, how do you know? And with Philly, with so many already people out of care, we just really didn’t feel like that was the best approach.” (Participant 2, Agency leader)
	Some clients did not feel comfortable performing self-testing procedures (e.g., finger stick for blood collection).	• “A lot of people are like, ‘I don’t want that.’ I want you to finger prick me. I want you to do it.’ We had to create a whole call center for the pandemic so that we can help people get [HIVST] kits. But people would just be like, ‘No. I don’t want that. That sounds too complicated.’” (Participant 3, Agency leader)
		• “I just had three weeks ago a person that was 14/15 years old, and she was so afraid of getting tested because she was afraid of the pin. So, I pinch myself and show the procedures and results. And [she] said, “Is that okay if I come back in an hour?” I said, “Of course.” And she came back after one hour because she felt that it was not easy. It’s a new thing for some people.” (Participant 5, HIV tester)

### Increased access to low-barrier HIV testing services

In general, HIV testers and agency leaders at publicly funded HIV testing agencies in Philadelphia viewed HIVST as a helpful strategy for increasing local HIV testing services. More than half of interviewees identified readily accessible publicly funded HIV testing kits as an important benefit for clients, and several described HIVST as a key resource for maintaining low-barrier testing services early in the COVID-19 pandemic. In our study, a participant reported that clients were requesting HIVST kits and sharing the kits they received with friends, family, and neighbors rather than completing self-testing themselves, which the participant suggested may have fortuitously extended HIV testing access. Interviewees described how helpful HIVST integration was early in the COVID-19 pandemic because it enabled their agencies to adapt existing testing programs to accommodate agency clients who either could not access in-person services or who preferred to avoid in-person services while continuing to meet the HIV testing metrics set by external funders.

### Accommodation for clients who prefer to avoid in-person venues

Several interviewees indicated they were supportive of HIVST integration because they felt HIVST increased testing among clients who were uncomfortable seeking traditional testing services in brick-and-mortar healthcare facilities that required the clients to interface closely with institutions they did not trust. They also reported some of their clients preferred more discrete testing methods that they could complete at home due to clients’ privacy concerns—more specifically, some clients were concerned they would be seen accessing services at a known HIV/STI testing location by other people in their community.

### Decreased risk of potential stigmatizing encounters

In addition to mitigating the community-level stigma clients experienced or anticipated they would experience when accessing in-person services, interviewees reported that providing HIVST kits was an appealing option for individuals who wanted to avoid HIV testing agencies due to prior negative experiences or anticipated medical mistreatment during in-person encounters when they interacted with healthcare workers. Several HIV testers in our study described how HIVST integration helped clients complete HIV testing without risking potential interpersonal stigma including stigmatizing attitudes and behaviors from clinicians, HIV testers, or other agency staff.

### Maintaining consistent connection with HIVST kit recipients

More than half of the staff interviewed in our study identified this potential loss of connection after self-testing as a key perceived barrier to HIVST integration at their agencies. One of the main concerns among both HIV testers and agency leaders was the lack of standard operating procedures for tracking individuals who received publicly funded HIVST kits and procedures for following up with kit recipients about their HIV test results. Notably, staff at agencies that had implemented standard operating procedures for following up with kit recipients expressed greater enthusiasm for HIVST integration than staff at agencies that lacked these procedures. One agency leader described how agency staff expressed concern about the potential loss of connection with their clients who received kits because staff were only mandated to collect a minimal amount of data about recipients. In response to this concern, the staff at the agency made a collective decision to develop procedures for consistently tracking recipients beyond what was required by external funders.

### Clients’ challenges correctly completing self-testing at home

HIV testers and agency leaders frequently expressed concerns about the potential loss of connection in conjunction with concerns that some clients may not complete at-home HIV testing correctly. Several interviewees were concerned that clients would use the kit incorrectly because many of the clients they serve had limited health literacy. One HIV tester expressed frustration that staff were not able to confirm if clients understood the written directions provided in the kit or able to confirm if the client conducted the test correctly because the agency did not have standard operating procedures for following up with kit recipients. Another HIV tester shared that even clients with sufficient health literacy may experience difficulty correctly interpreting at-home test results or may believe results are inaccurate.

### General client preference for in-person HIV testing services

More than half of interviewees were apprehensive about HIVST integration due to concerns that, unlike when clients who obtained on-site HIV testing, agencies might not know the test results or be able to link clients to HIV or PrEP services. One participant elucidated how the decision not to distribute HIVST kits was made at the agency level due to staff perception that rates of linkage and retention to HIV care were suboptimal among certain priority populations in Philadelphia. Interviewees also reported that some clients were reluctant to perform HIVST due to personal discomfort completing self-testing procedures such as using a lancet to puncture the skin and collect blood from a fingerstick.

### Mixed Methods Integration

We examined the extent to which our quantitative and qualitative findings were complementary or diverged when both methods were available for comparison. **Table 7** illustrates the results of the data integration and synthesis.

**Table 7:** Integration of main findings about HIV self-testing (HIVST) integration at publicly-funded HIV testing agencies in Philadelphia based on the original Consolidated Framework for Implementation Research (CFIR).

Quantitative Study	Qualitative Study	Interpretation and Synthesis
**CFIR DOMAIN: Intervention characteristics**		
Approximately two-thirds of respondents felt that HIVST would be simple to integrate into the HIV testing services at their respective agencies and into HIV testers’ routine duties (67%) and agreed that providing HIVST kits would ensure their clients regularly completed HIV testing (64%). However, 22% of respondents disagreed that providing HIVST kits would ensure clients regularly completed HIV testing and 14% of respondents disagreed that HIVST would be simple for clients to integrate into their lives as an HIV testing option.	There was consensus among HIV testers and agency leaders that HIVST was an HIV testing strategy that would be feasible to integrate into existing programs. However, three participants (including one agency leader) who approved of HIVST integration also discussed their experiences with clients who explicitly declined HIVST due to discomfort with the idea of at-home testing, needle aversion, or unsuccessful attempts to complete testing at home despite receiving guided instructions. Three HIV testers expressed concern that clients may perform the test or interpret the results incorrectly. No agency leaders discussed this topic.	In terms of adaptability and complexity, our results found that HIVST with accompanying telehealth sessions would be an acceptable and feasible HIV testing option to integrate into existing local HIV testing services including branded agency programming for the priority populations for which they tailored their services (e.g., LGBTQ + youth and/or adults; Black and Latine residents). However, our findings did not demonstrate a strong relative advantage of HIVST over traditional in-person testing services.
**CFIR DOMAIN: Outer setting**		
Approximately two-thirds of respondents believed that HIVST would improve clients’ access to HIV testing and reduce clients’ travel time to testing services. 60% of respondents believed that clients had an overall neutral experience when they received HIV counseling and testing via telehealth services. However, 43% of respondents reported technical/internet connectivity problems during telehealth sessions, and 7% of respondents perceived that clients’ telehealth experiences were somewhat or extremely negative.	More than half of the participants including two agency leaders felt that distributing HIVST kits increased access to HIV testing services among residents. Four participants including one agency leader discussed how HIVST was particularly appealing to their clients who wanted to complete HIV testing outside of either community-based or traditional healthcare settings for a variety of reasons (e.g., HIV-related stigma, medical mistrust, view home testing as more private option, convenience of taking test on their own schedule).	Our quantitative and qualitative findings suggested that the distribution of publicly funded HIVST kits may help agencies meet clients’ needs with respect to available low-barrier and geographically diverse HIV testing services, particularly in the context of limited client resources (e.g., limited funds for commercially available kits, time constraints, and transportation insecurity). HIVST may also help agencies meet the needs of clients that have experienced (or anticipate that they will experience) bias and discrimination when seeking HIV testing services.
Survey respondents were not asked about the ways in which the onset of the COVID-19 pandemic may have impacted HIV testing services at their respective agencies.	There was consensus among HIV testers and agency leaders that integrating HIVST into existing programs enabled agencies to reach more clients for testing in the context of pandemic restrictions and meet testing metrics set by external funders.	In terms of external policy and incentives, our qualitative analyses illustrated how the metrics set by external funders compelled several publicly funded HIV testing agencies to rapidly adapt existing HIV testing programs in response to widespread disruptions in traditional service delivery.
**CFIR DOMAIN: Inner setting**		
Approximately 70% of respondents felt HIVST would complement and was relevant to existing agency HIV prevention outreach activities. 60% of respondents reported that their agencies offered to mail HIVST kits to clients’ homes, and 64% of respondents reported they would encourage their agency clients to use HIVST. In addition, more than 70% of respondents reported that it was fairly easy or very easy to link clients to HIV medical care (72%) and PrEP care (77%) during a telehealth session.	More than half of participants (including two agency leaders) expressed concerns about the potential to lose contact or connection with clients after providing an HIV home test kit. Multiple participants described specific concerns that they would not be able to ensure kit recipients who had a reactive at-home test result to follow up confirmatory HIV testing and HIV medical care. Two participants including one agency leader expressed strong disinterest in HIVST integration or felt HIVST implementation was not appropriate at their agency due to concerns about perceived suboptimal linkage rates to local HIV medical services.	Quantitative and qualitative findings suggested that the organizational culture across more than half of agencies generally supported HIVST among clients, with variable degrees of readiness and enthusiasm for HIVST implementation. In terms of networks and connections, our quantitative findings indicated that respondents felt capable of linking clients to post-test services without significant difficulty. In contrast, our qualitative findings suggested that frontline staff and agency leaders shared notable concerns about suboptimal linkage. With respect to the implementation climate across agencies, concerns about linkage were not so substantial that the majority of participants would altogether discourage the use of HIVST among clients.
Approximately 80% of respondents were familiar with HIVST, felt that it would be easy to show clients how to use an HIVST kit during a telehealth session, and felt comfortable communicating with clients about using a kit.	Agency staff differed in readiness to provide HIVST telehealth services. There was consensus among HIV testers that testers at their agencies were knowledgeable about HIVST and had the self-capacity to discuss how to correctly use kits with clients, even among staff employed at agencies that did not distribute kits to clients. Those who had formal standard operating procedures, technical support (e.g., call centers), and other material resources described greater willingness to integrate HIVST into their programs. Those who perceived they were being asked to distribute kits without any clear guidance for following up with recipients refused to distribute kits to clients altogether.	Our quantitative findings showed high rates of familiarity with HIVST and comfort communicating with clients about HIVST. However, our qualitative findings revealed variable degrees of readiness for implementation across agencies. Testers whose agencies had HIVST-specific SOPs and were able to collaborate with other team members to troubleshoot issues as they arose reported greater self-efficacy to assess clients’ needs, maintain contact with kit recipients, and link clients to post-test services. However, several participants felt there was suboptimal agency-specific planning, insufficient available resources (e.g., standard operating procedures), and inadequate access to knowledge and information in the form of dedicated training in virtual HIV testing service delivery. Interviewees reported that many participating agencies addressed these issues over time.

The CFIR construct relevant to each interpretation is underlined in the interpretation and synthesis column. Based on the Consolidated Framework for Implementation Research by Damschroder and Lowery (*Implementation Science*, 2013) [37]

## Discussion

This work leveraged a community-academic implementation partnership to explore perceptions about integrating HIVST into HIV testing programs among frontline staff and agency leaders at publicly-funded HIV testing agencies that serve clients within an urban EHE priority jurisdiction in the United States. The triangulation and synthesis of our key quantitative and qualitative findings suggested that HIVST integration was acceptable to the frontline staff and agency leaders in our study and also highlighted determinants at the level of the system and agency that might be targeted to better support the uptake and widespread implementation of HIVST.

In terms of intervention characteristics, HIVST integration was an appealing testing option across multiple agencies among staff—many of whom had more than five years of experience working in the HIV treatment and prevention field. Agency staff in our study were familiar with HIVST, considered HIVST readily adaptable to fit existing programs tailored for a variety of client populations, and believed the technology itself was not prohibitively complex from a staff perspective, so staff felt comfortable instructing clients about at-home testing. However, concerns were raised about clients’ possible discomfort and difficulty completing at-home testing correctly even when clients received guided instructions or demonstrations during telehealth sessions. Although HIVST with accompanying telehealth services was felt to be acceptable and feasible among staff, our synthesis indicated that HIVST might not be an acceptable option to all clients and should be considered an adjunctive strategy rather than a replacement for traditional in-person testing services.

Given the success of the federally funded, direct-to-consumer national HIV self-test distribution program, which distributed over 400,000 HIVST kits in the first year of operation, the CDC renewed the call for clinicians, community organizations, and public health officials to consider including HIVST strategies in HIV testing programs [41]. Our synthesis indicated that although many staff in our study welcomed the opportunity to integrate HIVST into existing HIV testing programs, there were outer setting determinants of successful HIVST integration that warrant consideration. First, agency staff who participated in our study highlighted the importance of tailoring publicly funded HIVST programs to reach residents who are reluctant to interact with HIV testers in healthcare and community-based venues. Participants believed that while many clients were comfortable accessing in-person services, there was a substantive number of individuals in the community who would benefit from publicly funded HIVST distribution, particularly those for whom fear of experiencing potential HIV-related stigma was a strong deterrent to accessing in-person services in their community. These results were consistent with prior findings that some individuals who were interested in HIV testing prioritized discretion when they sought testing due to HIV-related stigma, particularly individuals with multiple marginalized identities who experience multiple axes of discrimination and oppression [13,18,25,42,43]. Second, participants in our study also recommended that health departments and community partners focus HIVST resources toward residents who might otherwise be interested in obtaining HIV testing but experienced persistent challenges due to limited time or material resources (e.g., clients who found commercially-available HIVST kits cost-prohibitive) or were not aware that HIV testing is a health behavior that is relevant for all sexually active people. The specific ways that health departments in the United States overcome these barriers will differ in response to the needs of the local priority populations and community partners. For example, several large-scale HIVST programs concentrated resources—in some cases almost exclusively—on increasing HIVST uptake among sexual and gender minority individuals assigned male sex at birth [17,24,41,44]. However, in response to the needs of trusted community partners, the Philadelphia Department of Public Health elected to distribute HIVST kits to residents of all genders and sexual orientations and iteratively refined its HIV home testing program services and media campaigns to broaden the program’s reach and ensure residents from other priority populations such as cisgender Black women were unequivocally included in its efforts to diagnose persons with HIV early [3]. Presenting HIV testing as a health protective behavior regardless of one’s identities or behaviors also aligns with the ongoing shift toward status-neutral approaches to HIV treatment and prevention services [7] and may be useful in reducing HIV testing-related stigma. Third, our qualitative analyses illustrated the degree to which performance measures set by external funders compelled many—but not all—publicly funded HIV testing agencies to adapt their testing programs early in the COVID-19 pandemic in response to widespread disruptions in traditional service delivery. Staff across multiple agencies described this time period as both a stress test and a unique opportunity to rapidly modify their programs with collective input from frontline staff and agency leadership. Agency leaders that elected to integrate HIVST further highlighted how rapid adaptation was critical to maintaining agency reach and viability, particularly among agencies highly reliant on public grant support. Collectively, our synthesis suggested that staff may be most enthusiastic about HIVST integration when external funders construct grants (and the performance metrics by which they measure success) in a way that affords trusted community partners greater flexibility to adapt rapidly programs with greater independence and when funders prioritize partners that are deeply committed to engaging residents in communities that have been difficult to engage via traditional HIV testing services or where in-person testing resources are limited.

In terms of inner setting determinants of HIVST integration, our synthesis indicated that the organizational cultures across many local publicly funded HIV testing agencies were generally supportive; however, important modifiable agency-level barriers to HIVST integration were noted. Our quantitative and qualitative findings diverged with respect to staff perceptions regarding linkage to post-test services. Our quantitative analyses found that agency staff felt it was fairly easy to link clients who completed HIVST to HIV and PrEP services. In contrast, our qualitative analyses revealed general concerns related to challenges following up with kit recipients and inconsistent processes for monitoring their testing outcomes across agencies, which made it difficult to estimate the degree to which staff were able to link self-testers to local post-test services successfully. Of note, the Philadelphia Department of Public Health found that the linkage rate to care within one month of diagnosis for individuals newly diagnosed with HIV in Philadelphia ranged from 81.3% in 2019 to 77% in 2022 [3], which may explain these qualitative findings. These findings also aligned with existing literature that has shown concerns about suboptimal post-test linkage remain a key barrier to HIVST uptake [22]. Importantly, in our study these concerns were attenuated by presence of agency-specific standard operating procedures for collecting recipient information and following up with recipients to discuss their testing experiences and results. Regrettably, evidence-based approaches for linking self-testers to post-test services are limited [16]. The Philadelphia Department of Public Health has been able to confirm if individuals who requested HIVST kits directly from the *Philly Keep on Loving* website subsequently engaged in HIV care in Philadelphia County via the Philadelphia Department of Public Health surveillance system; however; kits obtained via other methods may not be captured consistently [3]. To address this inner setting barrier, staff from several agencies in our study implemented new procedures to capture more detailed information from HIVST kit recipients (e.g., multiple methods of contact), which has enabled staff to follow up with recipients more consistently. In addition to serving as information clearinghouses and providing technical assistance, health departments should consider ways to leverage their knowledge regarding effective local strategies for post-test linkage to help agencies develop data-driven strategies that balance minimal data collection requirements to support low-barrier services and staff preferences for more comprehensive data collection to facilitate post-test linkage for each agency’s specific client population(s). Future research is needed to determine how best to develop strategies for creating low-barrier HIVST programs that optimize reach and successfully engage self-testers who receive reactive at-home test results to ensure they receive timely linkage to confirmatory HIV testing and care.

Lastly, large scale disruption of HIV testing services early in the COVID-19 pandemic adversely impacted HIV testing rates and linkage to necessary medical care [15]. In spite of these challenges, many publicly funded HIV testing agencies viewed this time as an opportunity to adapt programs in real time with enthusiasm and buy-in from frontline staff and agency leaders. In the era of persistent calls for austerity [45–48] and declining trust in public health institutions [49,50], local health departments may leverage lessons learned early in the COVID-19 pandemic to foster greater trust with community partners by providing more lenient funding parameters and performance metrics to promote innovation and empower partners as they develop culturally tailored services that bolster trust in public health institutions and progress in their shared goal of achieving health equity. Continuing HIVST programs may also help community partners maintain connections with residents located outside of agencies’ typical catchment areas and mitigate ongoing testing inequities in areas where in-person testing services are limited. In addition, although access to in-person services has been restored in many communities, interest for publicly funded kits mailed directly to residents’ homes early in the COVID-19 pandemic suggests that health departments may be able to better address residents’ HIV testing and other health needs using direct-to-home services as a way to create sustainable programs that reach a more geographically diverse population.

## Limitations

This study provided a nuanced exploration of the experiences and perceptions of HIVST among staff at publicly-funded HIV testing agencies; however, it has several limitations. First, the small sample size was limited to frontline staff (e.g., HIV testing and PrEP navigation positions) and agency leaders, many of whom were highly experienced in HIV service delivery in an urban and densely populated *EHE* priority jurisdiction that is allocated additional federal funding and resources to reduce HIV transmission. This limits the generalizability of our results to agencies with less experienced personnel, less funding, or fewer material resources. Future larger scale studies that include staff with lower levels of experience in HIV service delivery and agencies supported with alternative funding mechanisms or located in different settings (e.g., rural communities) are warranted. Second, this study examined perceptions among agency staff related to HIVST integration using publicly-funded kits. Programs that do not receive publicly funded kits (e.g., incurred expenses to obtain kits) may experience logistical and financial barriers to HIVST integration that were not captured in this study, and these potential barriers should be explored in future studies. Third, 30% of HIV testers and agency leaders who were invited to participate in interviews agreed to do so. Sociodemographic data collection for interview participants was limited to their place of employment and primary agency role/position to minimize the risk of potential confidentiality breaches. The goal of most qualitative studies is not to optimize statistical generalization but rather to provide a contextualized understanding of the phenomenon of interest [51,52]. However, it is important to note that the HIV testers and agency leaders who did not agree to interview in our study may have provided additional nuanced and insightful information, which may have improved the analytic generalization of our mixed methods study (i.e., the degree to which our findings are insightful, inductive generalizations) [52]. Finally, this study did not examine the experiences and perceptions of HIVST among HIVST recipients. Future research should include input from intended recipients of HIVST kits and other invested parties to confirm HIVST will be acceptable and salient to their needs and preferences before the HIVST program is implemented at other sites.

## Conclusion

In conclusion, this study added to existing literature by identifying system-level and agency-level barriers and facilitators to HIVST integration since the onset of the COVID-19 pandemic using a mixed-methods approach. The integration of our findings indicated HIVST was a viable strategy and generally viewed as favorable among this diverse group of staff at community-based HIV testing agencies including agency leaders. In addition, our findings highlighted the importance of bolstering community-based partnerships and the continued need for robust investment in resources to support efficient linkage to HIV-/PrEP-related care after self-testing. The system-level interventions that the health department is developing through this communityacademic partnership include strategies that are tailored to local contextual needs and diverse priority populations and reinforce the importance of equitable HIV testing practices. Eliminating structural barriers to testing among communities disproportionately impacted by HIV is critical to achieving population-level improvements in HIV status awareness, a key component of the Diagnose, Treat, and Prevent pillars of the national EHE Initiative in the United States. Integration of HIVST into the public health infrastructure has the potential to improve publicly funded efforts to implement more equitable HIV testing services.

## Supporting information

S1 File Quantitative survey. (PDF)

S2 File Semi-structured interview guide. (PDF)
