# Supplementary material for: Acceptability and feasibility of HIV self-testing integration into publicly-funded HIV prevention services: Perspectives from HIV testing agency staff that provide HIV testing services to sexual and gender minority youth in Philadelphia County
Source: PLoS One. 2025 Mar 25;20(3):e0320290. doi: 10.1371/journal.pone.0320290 (PMC11936223; doi:10.1371/journal.pone.0320290)
Supplement: S1 File — (PDF) [file pone.0320290.s001.pdf]

Thank you for agreeing to take this survey. We are asking HIV testers in our prevention system across all of our funded agencies a series of questions to identify opportunities for training and capacity building with the goals of fostering improved testing experiences for all people in Philadelphia, increasing linkages to HIV treatment and HIV prevention care, and building sustained professional development opportunities for front line workers in the HIV workforce. All answers will be confidential. There will be no punitive actions as a result of your responses, your agency's responses, or the responses of the overall Prevention system.

Demographics & Work Experience

What is your current age? __________

How do you identify your gender identity?

- Male
- Female
- Transgender man/Transmasculine
- Transgender female/Trans feminine
- Non-binary, gender non-conforming, or gender queer

How do you identify your sexual identity?

- Gay, lesbian, or homosexual
- Same-gender loving/Same-gender attracted
- Heterosexual
- Bisexual
- Asexual
- Pansexual
- Queer
- Other, please specify: ___________________

Do you consider yourself Hispanic or Latino?

- Yes
- No

What is your race? [select all that apply]

- American Indian or Alaskan Native
- Asian
- Black or African American
- Native Hawaiian or Other Pacific Islander
- White or Caucasian
- Other, specify: _________________

Do you consider yourself Middle Eastern?

- Yes
- No

Which of the following organizations/agencies do you currently work or volunteer for? [If more than 1, choose your primary location.]

- List of organizations funded for HIV prevention services by the City of Philadelphia Department of Public Health [drop-down menu]
- Other → Ineligible

Which of the following describes your current position with this organization/agency? [if more than 1, choose your primary position.]

- Administrative staff (e.g., receptionist, finances) → Ineligible
- HIV Test Counselor (*skip pattern*: if marked, go to 2a.)
- HIV Testing Supervisor/Lead HIV Tester

- PrEP Navigator
- Leadership staff (e.g., CEO, president, program director) → Ineligible

Are you currently certified by the Commonwealth of Pennsylvania to provide HIV test counseling services?
- Yes
- No → Ineligible
- I don't know. → Ineligible

Which of the following best describes your current position?
- I am a full-time (32 hours or more per week) salaried employee at my organization/agency.
- I am a part-time salaried (less than 32 hours per week) employee at my organization/agency.
- I am an hourly paid employee at my organization/agency.
- I am a volunteer worker at my organization/agency.

How long have you served in your primary role at your organization/agency?
- 0-3 months
- 3-5 months
- 6-11 months
- 12 months or more

How long have you been an HIV tester?
- Less than 1 year
- 1-2 years
- 3-4 years
- 5-7 years
- 8-10 years
- 11-15 years
- 15+ years

How long have you been working in the field of HIV services?
- Less than 1 year
- 1-2 years
- 3-4 years
- 5-7 years
- 8-10 years
- 11-15 years
- 15+ years

Thinking about your experiences as a HIV tester to date, please reflect on your ability to do the following activities during a HIV Testing visit:

Explain client confidentiality before HIV testing
- Poor
- Fair
- Good
- Excellent

Help a client cope with their HIV diagnosis if their HIV test is positive
- Poor
- Fair
- Good
- Excellent

Immediately Link a client to HIV Care if their HIV test is positive

- Poor
- Fair
- Good
- Excellent

Explore whether HIV Pre-Exposure Prophylaxis (PrEP) is right for the client
- Poor
- Fair
- Good
- Excellent

Discuss benefits and risks of HIV Post-Exposure Prophylaxis (PEP) with a client
- Poor
- Fair
- Good
- Excellent

Offer condoms and lubricant (as appropriate) to a client
- Poor
- Fair
- Good
- Excellent

Refer people who are using drugs (e.g., Opioids. Injection drug use) to Naloxone and Syringe Access Programs
- Poor
- Fair
- Good
- Excellent

Refer a client to test for other STI services, including STI testing and treatment.
- Poor
- Fair
- Good
- Excellent

Explore clients' reasons for testing if they identify as cisgender women
- Poor
- Fair
- Good
- Excellent

Explore clients' reasons for testing if they are people who inject drugs
- Poor
- Fair
- Good
- Excellent

Explore clients' reasons for testing if they identify as transgender or non-binary
- Poor
- Fair
- Good
- Excellent

Explore clients' reasons for testing if they identify as men who have sex with men

- Poor
- Fair
- Good
- Excellent

Use of Rapid HIV Home Tests

Have you ever heard about rapid home HIV test kits?

__ Yes
__ No

Does your agency offer clients the option to receive rapid home HIV test kits at home?

__ Yes
__ No
__Don’t Know

How familiar are you with rapid HIV home test kits?

1. Not at all familiar
2. Vaguely familiar
3. Somewhat familiar
4. Very familiar

How useful do you think rapid HIV home test kits will be if implemented as a complement to your agency’s existing HIV testing service capacity?

1. Not at all useful
2. Somewhat useful
3. Very useful
4. Extremely useful

What relevance do you see for rapid HIV home test kits as related to HIV Prevention outreach activities conducted at your agency?

1. Not at all relevant
2. Somewhat relevant
3. Very relevant
4. Extremely relevant

Compared to in-person HIV testing, how likely would you be to encourage clients to use an over-the-counter HIV test?

There is no way I would encourage the use of a rapid HIV home test kit to test my clients.

- Strongly Agree
- Agree
- Neutral
- Disagree
- Strongly Disagree

It is unlikely that I would encourage the use of a rapid HIV home test kit to test my clients.

- Strongly Agree
- Agree
- Neutral
- Disagree
- Strongly Disagree

It is likely that I would encourage the use of a rapid HIV home test kit to test my clients.

- Strongly Agree

- Agree
- Neutral
- Disagree
- Strongly Disagree

I would definitely encourage the use of a rapid HIV home test kit to test my clients.

- Strongly Agree
- Agree
- Neutral
- Disagree
- Strongly Disagree

Please indicate how *hard* or *easy* it would be for you to do each of the following things:			
1<br>very easy to do	2<br>fairly easy	3<br>fairly hard	4<br>very hard to do

Send rapid HIV tests to clients' home.

Guide a client through a HIV testing and PrEP referral visit through a phone or video visit (telehealth).

Ask a client to take a rapid HIV test through a telehealth visit.

Show a client how to use a rapid HIV test through a telehealth visit.

Complete post-test counseling on HIV prevention during a telehealth visit

Link a patient to HIV care during a telehealth visit

Link a patient to PrEP care during a telehealth visit.

Usefulness

A rapid home HIV test kit will improve clients' access to HIV prevention services.

- Strongly Agree
- Agree
- Neutral
- Disagree
- Strongly Disagree

A rapid home HIV test kit will save clients' time traveling to HIV prevention services.

- Strongly Agree
- Agree
- Neutral
- Disagree
- Strongly Disagree

A rapid home HIV test kit provides for clients' healthcare needs.

- Strongly Agree
- Agree
- Neutral
- Disagree
- Strongly Disagree

Ease of Use & Learnability

A rapid home HIV test kit will be simple to integrate into my agency's testing capacity.

- Strongly Agree
- Agree
- Neutral
- Disagree
- Strongly Disagree

A rapid home HIV test kit will be simple to integrate into my responsibilities as a test counselor.
- Strongly Agree
- Agree
- Neutral
- Disagree
- Strongly Disagree

A rapid home HIV test kit will be simple for clients to integrate as an HIV testing option.
- Strongly Agree
- Agree
- Neutral
- Disagree
- Strongly Disagree

Satisfaction, Acceptability, and Future Use

I would feel comfortable communicating with a client about using a rapid home HIV test kit.
- Strongly Agree
- Agree
- Neutral
- Disagree
- Strongly Disagree

A rapid home HIV test kit is an acceptable way to receive an HIV test.
- Strongly Agree
- Agree
- Neutral
- Disagree
- Strongly Disagree

A rapid home HIV test kit will ensure that clients get tested regularly.
- Strongly Agree
- Agree
- Neutral
- Disagree
- Strongly Disagree

I am excited about a rapid home HIV test kit] as an HIV testing strategy for my clients.
- Strongly Agree
- Agree
- Neutral
- Disagree
- Strongly Disagree

Comprehensive Sexual Health Provision

Please indicate your agreement or disagreement with each statement below.
(1=Strongly disagree; 6=Strongly agree).

Discussing sexuality is essential to client's health outcomes.

I understand how my clients' health and treatments might affect their sexuality.

I am uncomfortable talking about sexual issues.

I am more comfortable talking about sexual issues with my clients than are most of the staff I work with.

I make time to discuss sexual concerns with my clients.

Whenever clients ask me a sexuality-related question, I advise them to discuss the matter with a physician.

I feel confident in my ability to address clients' sexual concerns.

Sexuality is too private an issue to discuss with clients.

Giving a client permission to talk about sexual concerns is part of my work responsibilities.

Sexuality should be discussed only if initiated by the client.

Clients expect HIV testing staff to ask about their sexual problems.

Expanded sexual health services will benefit clients.

Which of the following are barriers that you experience when discussing sexual health promotion with a client? Mark all that apply.

1. Not enough time
2. The presence of a third party (another person who is not the client)
4. Getting high-risk groups to attend
5. Training not a major priority
6. Lack of knowledge/training
7. Language/ethnicity issues
8. Lack of financial incentives
9. Knowing the client outside of the agency
10. Issues relating to a small community
11. Client's gender identity
12. Cultural/religious issues
13. Embarrassment
14. Issues relating to sexuality
15. Age difference between me and the client
16. Fear of uncovering a difficult problem
17. Other; please specify

When a clinic practices patient-centered care:
- Medical providers are solely responsible for practicing it.
- All staff members are responsible for practicing it.
- A certification is required by their state's health department.
- Both B and C

When taking a sexual health history with LGBTQ+ youth, it is best to:
- Use open-ended questions.
- Tell them they are high risk.
- Use normalizing language.
- Both A and C

Which of the following is the best way to start a conversation about STI prevention with a client?

- “I’d like to talk for a minute about preventing STIs and what you think will work best for you. Is that alright with you?”
- “You are what we consider high risk, and we really need to talk about that today, okay?”
- “Do you think using condoms more often will work for you? What about limiting your number of sexual partners or using PrEP?”
- “Talk to me about why condoms don’t work for you.”

Tester knowledge of expanded sexual health services, including rapid HIV and HCV testing, STI testing, and linkage to PrEP, PEP and treatment (HIV, HCV, and STI)

For each statement below, please circle true (T), false (F), or I don’t know (DK). If you don’t know, please do not guess; instead, please circle DK

Genital herpes is caused by the same virus as HIV.	T	F	DK
There is a cure for Gonorrhea.	T	F	DK
It is easier to get HIV if a person has another sexually transmitted infection.	T	F	DK
Human Papillomavirus (HPV) is caused by the same virus that causes HIV.	T	F	DK
Having anal sex increases a person’s risk of getting Hepatitis B.	T	F	DK
Soon after infection with HIV a person develops open sores on his or her genitals (penis or vagina).	T	F	DK
There is a cure for Chlamydia.	T	F	DK
The same virus causes all of the sexually transmitted infections.	T	F	DK
Human Papillomavirus (HPV) can cause Genital Warts.	T	F	DK
Using a natural skin (lambskin) condom can protect a person from getting HIV.	T	F	DK
Sexually transmitted diseases can lead to health problems that are usually more serious for men than women.	T	F	DK
If a person tests positive for HIV the test can tell how sick the person will become.	T	F	DK
There is a vaccine available to prevent a person from getting Gonorrhea.	T	F	DK
A person who has genital herpes must have open sores to give the infection to his or her sexual partner.	T	F	DK
There is a vaccine that prevents a person from getting Chlamydia.	T	F	DK
If a person had Gonorrhea in the past, he or she is immune (protected) from getting it again.	T	F	DK
Human Papillomavirus (HPV) can cause HIV.	T	F	DK
There is a vaccine that can protect a person from getting Hepatitis B.	T	F	DK

Additional items for HCV

Studies show that 60% of people who inject street drugs are infected with hepatitis C.

- True
- False
- Don’t Know

People can live with hepatitis C for many years without knowing that they have been infected with the virus.

- True
- False
- Don’t Know

Hepatitis C can be given to someone during sexual intercourse.
- True
- False
- Don't Know

Once someone's hepatitis C virus has been completely treated and cleared, that person cannot get re-infected with hepatitis C.
- True
- False
- Don't Know

PrEP Service Provision Behavioral Skills

Determining whether a client's sexual risk behaviors warrant the use of PrEP.
- Completely uncomfortable
- Uncomfortable
- Neutral
- Comfortable
- Completely comfortable

Determining whether a client's drug use behaviors warrant the use of PrEP.
- Completely uncomfortable
- Uncomfortable
- Neutral
- Comfortable
- Completely comfortable

Discussing the effectiveness of PrEP with a client.
- Completely uncomfortable
- Uncomfortable
- Neutral
- Comfortable
- Completely comfortable

Discussing whether PrEP is a good option for a client.
- Completely uncomfortable
- Uncomfortable
- Neutral
- Comfortable
- Completely comfortable

Working with clients to identify a PrEP provider.
- Completely uncomfortable
- Uncomfortable
- Neutral
- Comfortable
- Completely comfortable

Assisting clients set up medical appointments with a PrEP provider.
- Completely uncomfortable
- Uncomfortable
- Neutral
- Comfortable

- Completely comfortable

Discussing how to navigate barriers to accessing medical appointments with PrEP providers.

- Completely uncomfortable
- Uncomfortable
- Neutral
- Comfortable
- Completely comfortable

PrEP Stigma

People taking PrEP are being reckless.

- Strongly Agree
- Agree
- Neutral
- Disagree
- Strongly Disagree

People taking PrEP are being responsible.

- Strongly Agree
- Agree
- Neutral
- Disagree
- Strongly Disagree

People taking PrEP are helping to prevent HIV.

- Strongly Agree
- Agree
- Neutral
- Disagree
- Strongly Disagree

People taking PrEP are protecting themselves.

- Strongly Agree
- Agree
- Neutral
- Disagree
- Strongly Disagree

People taking PrEP are putting their partners at risk.

- Strongly Agree
- Agree
- Neutral
- Disagree
- Strongly Disagree

People taking PrEP are undermining condom use.

- Strongly Agree
- Agree
- Neutral
- Disagree
- Strongly Disagree

I support people taking PrEP.
- Strongly Agree
- Agree
- Neutral
- Disagree
- Strongly Disagree

Work Environment

The following questions ask about your clinic as a whole. If you don't know whether or not your clinic does or has done any of the following, please answer "Don't know."

Our clinic has educational materials specifically tailored for gay or bisexual young men.
- Yes
- No
- Don't know

Our clinic has educational materials specifically tailored for transgender and gender nonconforming youth.
- Yes
- No
- Don't know

Our clinic has affirming posters for LGBTQ+ youth.
- Yes
- No
- Don't know

Our clinic has a referral list that includes resources for gay and bisexual young men.
- Yes
- No
- Don't know

Our clinic has a referral list that includes resources for transgender and gender nonconforming youth.
- Yes
- No
- Don't know

Our clinic has a policy that allows all people to use the restroom that is consistent with their gender identity.
- Yes
- No
- Don't know

Our clinic has a single occupancy restroom that is gender neutral or unisex.
- Yes
- No
- Don't know

Our clinic has a non-discrimination policy that includes gender identity and gender expression.
- Yes
- No
- Don't know

Our clinic has a non-discrimination policy that includes sexual orientation.
- Yes
- No

- Don't know

Overall, I think our clinic is a safe space for gay and bisexual young men.

- Yes
- No
- Don't know

Overall, I think our clinic is a safe space for transgender and gender nonconforming youth.

- Yes
- No
- Don't know

I know where I can find additional resources to better serve LGBTQ+ youth.

- Yes
- No
- Don't know

I am willing to work with my co-workers to create a more affirming clinic for transgender and gender nonconforming youth.

- Yes
- No
- Don't know

I am willing to work with my co-workers to create a more affirming clinic for gay and bisexual young men.

- Yes
- No
- Don't know

Feel Safe

I feel safe on the job [when providing HIV test counseling].

- Strongly Agree
- Agree
- Neutral
- Disagree
- Strongly Disagree

Working Remote/Telehealth

How did you professionally prepare for transitioning HIV test counseling via teleconference?

- Spoke to colleagues
- Read guidelines from the health department
- Read guidelines from my agency
- Attended online trainings/webinars
- Meetings with my supervisor
- Not prepared specifically
- I had used teleconference platforms in other parts of my job
- I had used teleconference platforms in prior or other jobs
- Other, please specify

What are the main challenges for you conducting HIV test counseling via teleconference?

- Technical/Internet connectivity problems
- Difficult for client to find a private, suitable space
- Risk of client getting distracted

- Difficulty feeling connected with client
- Risk of myself getting distracted
- Difficulty to find a private, suitable space for myself
- Confidentiality concerns
- Difficulty keeping professional boundaries
- Other, please specify

Compared to in-person HIV counseling and testing, online sessions make me feel…
- Less authentic/genuine
- Just as authentic/genuine
- More authentic/genuine

Compared to in-person HIV counseling and testing, online sessions make me feel _________ to do my job.
- Less confident
- Just as confident
- More confident

How do perceive your clients' experiences with telehealth HIV counseling and testing?
- Extremely negative
- Somewhat negative
- Neutral
- Somewhat positive
- Extremely positive

Profession Adaptation ("Good Soldiering")

I believe that the positive aspects of my job outweigh the negative aspects.
- Strongly Agree
- Agree
- Neutral
- Disagree
- Strongly Disagree

I believe that working in [HIV prevention] is important work.
- Strongly Agree
- Agree
- Neutral
- Disagree
- Strongly Disagree

Do you think that these rewards decrease the stress that you experience on the job?
- Yes
- No

Thank you for completing the survey; we appreciate your time. We will be providing ongoing capacity building and training opportunities for the HIV prevention work force based on the identified needs.
