# Supplementary material for: Acceptability and feasibility of HIV self-testing integration into publicly-funded HIV prevention services: Perspectives from HIV testing agency staff that provide HIV testing services to sexual and gender minority youth in Philadelphia County
Source: PLoS One. 2025 Mar 25;20(3):e0320290. doi: 10.1371/journal.pone.0320290 (PMC11936223; doi:10.1371/journal.pone.0320290)
Supplement: S2 File — (PDF) [file pone.0320290.s002.pdf]

**<u>Interview Guide: HIV Test Counselors and PrEP Navigators</u>**

Before we start, I want to thank you again for your participation in this study. Your participation may contribute to a better understanding of the organizational factors within your agency that may shape the integration of Pre-Exposure Prophylaxis, commonly known as PrEP, into existing prevention services. By organizational factors, we mean dynamics within your agency that you believe shape service provision and may in turn affect how PrEP services are implemented. I want to remind you that everything we talk about today will be completely confidential. You may speak freely about your thoughts, opinions, and experiences. We will be recording this interview; however, the audio file will only be used by the research team and myself. To protect your privacy and that of others you may mention, we will not ask identifying questions about the people or organizations you talk about. Please use only a first name or alias when talking about a specific person. We expect this interview to run between 45 minutes to an hour and we can take a break if needed. Remember, if you ever feel uncomfortable or do not want to answer any of these questions, we can pause or stop the interview at any time. Before we begin, do you have any questions?

<u>Professional Role</u>:
Ok. Let's start by talking about your professional role in Philadelphia.

1. Can you describe for me your professional role at (name of organization)? And how long have you been in this position?

2. To what extent and in what capacity do you interact with young sexual and gender minorities (SGM)? By young SGM, I am talking about young men and transgender women who engage in oral or anal sex with men and who are between the ages of 15 and 29 years of age.

3. To what extent do you network with colleagues or people in similar professions/positions outside of your agency?

4. To what extent does your organization encourage you to network with colleagues outside your own setting?

<u>Agency Role in HIV Prevention</u>:

1. From your perspective, how would you describe the HIV/AIDS epidemic in the city of Philadelphia?
    - *How would you describe the epidemic specifically among young* SGM *in Philadelphia?*
    - *How does the epidemic differ specifically within young* SGM *and other populations in Philadelphia?*

<u>Current Prevention Services</u>:
Now we're going to talk a bit more specifically about the services that your agency provides.

1. Can you describe for me prevention services that your organization currently offers/promotes?

2. What are some of the obstacles that stop young SGM from getting access to your agency's services?

3. Does your agency currently offering STI testing services?
    - {if Yes} In your opinion, what resources have made the implementation of STI testing services successful at your agency?
    - {If Yes} What, if any, challenges or limitations has your agency experienced when offering STI testing at your agency?
    - {If No} Has your agency ever delivered, or thought about offering, STI testing services to clients? Tell me more about that.

4. How would the delivery of rapid HIV home test kits support HIV testing efforts in Philadelphia?
    - *What, if any, advantages do you see to the large-scale implementation of rapid HIV home test kits across Philadelphia County?*

- *In your opinion, what challenges or limitations would the implementation of rapid HIV home test kits have on your agency?*

5. In your opinion, do you feel that the staff in your agency are well-equipped to offer comprehensive sexual health services to clients?
   - *What strategies and resources have you leveraged to capacitate your staff in HIV service delivery?*
   - *What strategies and resources have you leveraged to capacitate your staff in STI service delivery?*
   - *What resources would help your agency increase its capacity to deliver comprehensive sexual health services?*

<u>PrEP Service Provision</u>:
The advances in biomedical prevention in HIV that have emerged in 2012 have medicalized parts of our HIV prevention and care delivery system.

1. Thinking back over the past 8 years, how has the medicalization of HIV prevention affected your agency's mission and HIV prevention service delivery?

2. What is your overall perception about how young SGM have responded to PrEP in Philadelphia?
   - *What are their general attitudes about PrEP?*

<u>Provider Insights</u>:

1. Have you had clients, specifically young SGM, inquire about PrEP? If so, can you tell me about what these conversations look like?
   - *What concerns, if any, did they have about starting PrEP?*

2. How do you decide if you are going to discuss PrEP with a young SGM client?
   - *How do you broach conversations about PrEP with your young MSM clients?*
     - *What do these conversations look like?*

3. Within your organization, have you or your colleagues who provide HIV prevention services ever encountered any issues in providing PrEP services to young SGM? If so, how did you/they go about addressing these issues?

4. Given all of what you know about PrEP service delivery, in your opinion, how should PrEP optimally be implemented in Philadelphia?

<u>Non-organizational Factors</u>:
Ok, so now we're going to discuss your thoughts and opinions of PrEP access outside of your organization.

1. What are some of the obstacles that may stop young SGM from accessing PrEP in Philadelphia?

2. What resources/assets are available in Philadelphia that might encourage young SGM to access PrEP?

3. What do you feel is unique, if anything, about Philadelphia that could facilitate young SGM's PrEP access and engagement in PrEP care.

<u>Conclusion</u>:
Okay, thank you for all this very insightful information. For this last section, I just have a few final questions about the interview.

1. Is there anything that we haven't asked you that you think we should have?

2. Anything that you feel is missing or should have been asked about in a different way?

**3.** Any other questions or comments?

**Interview Guide – Directors and Officials**

Before we start, I want to thank you again for your participation in this study. Your participation may contribute to a better understanding of the organizational factors within your agency that may shape the integration of Pre-Exposure Prophylaxis, commonly known as PrEP, into existing prevention services. By organizational factors, we mean dynamics within your agency that you believe shape service provision and may in turn affect how PrEP services are implemented. I want to remind you that everything we talk about today will be completely confidential. You may speak freely about your thoughts, opinions, and experiences. We will be recording this interview; however, the audio file will only be used by the research team and myself. To protect your privacy and that of others you may mention, we will not ask identifying questions about the people or organizations you talk about. Please use only a first name or alias when talking about a specific person. We expect this interview to run between 45 minutes to an hour and we can take a break if needed. Remember, if you ever feel uncomfortable or do not want to answer any of these questions, we can pause or stop the interview at any time. Before we begin, do you have any questions?

Professional Role:

1. Can you describe for me your professional role at (name of organization)? And how long have you been in this position?

2. What made you decide to pursue this position?

Agency Role in HIV Prevention:

1. From your perspective, how would you describe the HIV/AIDS epidemic in the city of Philadelphia?
   - *How would you describe the epidemic specifically among young sexual and gender minorities in Philadelphia?*
   - *How does the epidemic differ specifically between young sexual and gender minorities and other populations in Philadelphia?*

2. What have been your primary resources for getting information about the HIV epidemic?
   - *How do you keep up with new information and new technologies regarding the HIV epidemic?*

3. How do you see the contributing role of your agency regarding the HIV epidemic the Philadelphia area?
   - *What is the mission of your agency?*
   - *What are the services or resources that your agency is most known for providing?*
   - *What are the most common resources that your agency provides (or the most common that clients access and use at your agency)?*
   - *How does your agency support clients' access to comprehensive sexual health services?*

4. Does your agency currently offering STI testing services?
   - {If Yes} In your opinion, what resources have made the implementation of STI testing services successful at your agency?
   - {If Yes} What, if any, challenges or limitations has your agency experienced when offering STI testing at your agency?
   - {If No} Has your agency ever delivered, or thought about offering, STI testing services to clients? Tell me more about that.

5. How would the delivery of rapid HIV home test kits support HIV testing efforts in Philadelphia?
   - *What, if any, advantages do you see to the large-scale implementation of rapid HIV home test kits across Philadelphia County?*
   - *In your opinion, what challenges or limitations would the implementation of rapid HIV home test kits have on your agency?*

6. From your perspective, how connected is your agency to larger health and social service systems in Philadelphia?
   - *What has facilitated connections to these systems?*
   - *What has impeded your agency's ability to maximize the connections to these systems?*
   - *What resources would help you grow these relationships further?*

7. In your opinion, do you feel that the staff in your agency are well-equipped to offer comprehensive sexual health services to clients?
   - *What strategies and resources have you leveraged to capacitate your staff in HIV service delivery?*
   - *What strategies and resources have you leveraged to capacitate your staff in STI service delivery?*
   - *What resources would help your agency increase its capacity to deliver comprehensive sexual health services?*

<u>PrEP Service Provision</u>:
The advances in biomedical prevention in HIV that have emerged in 2012 have medicalized parts of our HIV prevention and care delivery system.

1. Thinking back over the past 8 years, how has the medicalization of HIV prevention affected your agency's mission and HIV prevention service delivery?

2. Generally speaking, where and when did you learn about PrEP?

3. As an HIV prevention services provider, what are your beliefs and attitudes about PrEP as a method for HIV prevention?
   - *How effective, if at all, do you believe PrEP is in preventing HIV?*
   - *In general, do you see PrEP as something negative or positive? What reasons do you see PrEP as [negative/positive]?*
     - *To what extent do you view PrEP as something negative or positive for young MSM?*
   - *How, if at all, have your beliefs changed from when you first heard about PrEP?*

4. What is your overall perception about how young sexual and gender minorities have responded to PrEP in Philadelphia?
   - *What are their general attitudes about PrEP?*

5. What is your overall perception about how agencies in Philadelphia County that serve young sexual and gender minorities have responded to PrEP?
   - *What are their general attitudes about PrEP?*
   - *Based on your perceptions, how, if at all, do your beliefs about PrEP align with how your organization views PrEP?*

Here is a list of possible PrEP-related services. [Present list.]

| | |
|---|---|
| Creating or increasing clients' PrEP awareness | Assisting clients to make an appointment with PrEP provider |
| Educating clients about PrEP | Providing PrEP medical care |
| Assessing clients for PrEP eligibility | Assisting clients adhere to PrEP regimen |
| Assisting clients locate a PrEP provider | |

6. Which of the services listed, if any, does [your organization/organizations in your jurisdiction] provide?
   - *How were decisions made in identifying which PrEP-related services would and would not be implemented?*

7. In thinking of the PrEP-related services offered by [your organization/jurisdiction], what do you believe are some, if any, structural or social environmental factors that hinder PrEP implementation? By structural, I'm referring to things like local or state policies and by social environmental factors, I'm referring to cultural factors and social norms around PrEP or HIV in general.

8. Based on your answers, how do you think city and/or state officials would react if we proposed policy changes to address some of these structural or social environmental factors?
   - *What do you foresee might be some barriers to implementing such changes?*
   - *What do you foresee might be factors that would facilitate such changes?*

9. In thinking of PrEP-related services offered by [your organization/jurisdiction] for young sexual and gender minorities, what do you believe are some organizational factors that hinder PrEP implementation. By organizational factors, I'm referring to factors related to staffing, training, resources and programming.
   - *Which resources, if any, are either missing or insufficiently supported, that if addressed may have a major impact in facilitating PrEP service provision?*

10. In thinking about PrEP services in [your agency/jurisdiction], what do you believe have contributed to successes in PrEP implementation for young sexual and gender minorities?

11. Describe for me any surveillance or evaluation infrastructure that currently exists to assess the progress of [your organization's/organizations in your jurisdiction's] PrEP service implementation and outcomes.

12. Based on the capacity of [your agency/the agencies in your jurisdiction], how feasible would it be to implement PrEP medical services versus referral/linkage systems?
    - *What factors would assist in increasing the feasibility of either of these services?*

13. What services are missing in Philadelphia County at large that you think could facilitate PrEP implementation for young sexual and gender minorities?

<u>Conclusion:</u>
Okay, thank you for all this very insightful information. For this last section, I just have a few final questions about the interview.

1. Is there anything that we haven't asked you that you think we should have?

2. Anything that you feel is missing or should have been asked about in a different way?

3. Any other questions or comments?

Okay, that concludes the interview. Thank you for your time.
